# Use of Geospatial Surveillance and Response Systems for Vector-Borne Diseases in the Elimination Phase

**DOI:** 10.3390/tropicalmed4010015

**Published:** 2019-01-18

**Authors:** John B. Malone, Robert Bergquist, Moara Martins, Jeffrey C. Luvall

**Affiliations:** 1 Pathobiological Sciences, School of Veterinary Medicine, Louisiana State University, Baton Rouge, LA 70803, USA; moaramartins@gmail.com; 2 Ingerod, SE-454 94 Brastad, Sweden; robert.bergquist@outlook.com; 3 National Aeronautics Space Administration (NASA), MSFC ST11, NSSTC, 320 Sparkman Drive, Huntsville, AL 35805, USA; jluvall@nasa.gov

**Keywords:** GIS, remote-sensing, satellite, international space station, ECOSTRESS, worldview, spatio-temporal epidemiology, climate change, parasite, schistosomiasis, leishmaniasis

## Abstract

The distribution of diseases caused by vector-borne viruses and parasites are restricted by the environmental requirements of their vectors, but also by the ambient temperature inside the host as it influences the speed of maturation of the infectious agent transferred. The launch of the Soil Moisture Active Passive (SMAP) satellite in 2015, and the new ECOSTRESS instrument onboard the International Space Station (ISS) in 2018, established the leadership of the National Aeronautics Space Administration (NASA) in ecology and climate research by allowing the structural and functional classification of ecosystems that govern vector sustainability. These advances, and the availability of sub-meter resolution data from commercial satellites, contribute to seamless mapping and modelling of diseases, not only at continental scales (1 km^2^) and local community or agricultural field scales (15–30 m^2^), but for the first time, also at the habitat–household scale (<1 m^2^). This communication presents current capabilities that are related to data collection by Earth-observing satellites, and draws attention to the usefulness of geographical information systems (GIS) and modelling for the study of important parasitic diseases.

## 1. Introduction

Disease and location were already linked by Hippocrates in the fourth century before the Christian era, and Snow famously traced a cholera outbreak to a particular water pump in London in the mid-1800s. Interestingly, this was before anybody had any idea about bacteria and viruses, even though van Leeuwenhoek probably saw the former in his rudimentary microscope, 170 years before Snow’s findings. However, some decades later, communicable diseases and their propagation were already quite well understood, but what was now missing was the technology needed to translate the data collected over large areas into reliable risk maps. Remote sensing (RS), first from airplanes, since the 1970s from satellites, and very recently also from drones, changed all that. RS became the impetus to the merger of Earth sciences, computer technology, and advanced statistics eventually granting access to an array of advanced tools that are highly suitable for epidemiological investigation [1,2,3,4]. Such techniques are particularly useful for the study of those parasitic infections that rely on intermediate hosts (vectors) to complete their life cycles, since these vectors (commonly insects but also molluscs) are highly sensitive to a range of environmental variables. In addition, temperature also limits the maturation of the parasite’s intermediate stage(s) inside the vector which, together with other variables, makes it possible to estimate disease distributions with a good level of accuracy. Indeed, the number of publications promoting the use of remotely sensed variables, such as land use, temperature, rainfall, humidity, vegetation etc., to effectively decide the distributions of infectious microorganisms, is increasing exponentially, as exemplified by Rogers and Randolph [5], Foley [6], Bergquist [7], Lord et al. [8], Misslin et al. [9], and a multitude of other authors. Temperature plays a major role for vector presence, and climate change has by now made it possible for diseases to start expanding their endemic areas, or can be expected to do so. For example, several tick-borne infections around the world [10], dirofilariasis in northern Europe [11], and schistosomiasis in northern China [12,13] are currently on the move. Pollution, and resistance to pesticides and drugs, as well as the general fall-out from globalization, are other factors driving such changes [14]. Previously locally confined infections that have lately become wide-spread are becoming more numerous on a monthly basis, e.g., the parasite *Babesia*, the spirochete *Borrelia*, and viruses such as chikungunya, Rift Valley virus (RFV), West Nile virus (WNV), and the Zika virus, to mention the most well-known [15]. 

## 2. Data Collection

Advanced laptop computers and widespread access to the Internet have created a broad accessibility of RS data from Earth-observing satellites. While this has made epidemiology more dependent on satellite-generated data, the discipline has also undergone a paradigmatic change, thanks to geographical information systems (GIS) that facilitate the management and processing of epidemiological data. The stronger potential to match the suitability of various environments to parasite life cycles and transmission dynamics provides a new way to address the nidality concept introduced by Pavlovskii as far back as 1945 [16]. Based on his ideas, geography and environmental variables associated with health data have led to the concept of disease ecology, where RS provides useful insights on the different factors related to transmission levels and disease distributions, while mapping and modelling facilitates interpretation, synthesis, and recognition of outbreak frequencies [17].

The GIS approach supports overlay and network analysis by documenting neighborhoods, buffers and spatial parameters, and today’s epidemiologists have access to a multitude of ecological and climatic data that were never before available in such amounts and with such ease. The visualization of epidemiological datasets in a geographical context, e.g., linking spatial data from virtual globes with GIS software packages supports prediction and risk profiling [18], while sharing epidemiological data in real-time, is helpful, not only for individual researchers, but also for decision-makers. The growth of the Internet has distributed GIS widely, connecting with other platforms, such as web map servers, libraries, spatial database management systems, and software development frameworks. The field has thus become multi-participatory, such as allowing the advantage of cloud computing opportunities that facilitate GIS access for anyone, anywhere. However, while the development of near real-time surveillance systems, based on GIS, global positioning systems (GPS) and RS, facilitate the establishment of accurate, up-to-date early-warning systems (EWS), it is important to understand that GIS neither makes the actual field collection of parasites and vector easier, nor does it assure the quality of the information gained [19,20].

In the published literature on health applications of the geospatial sciences since the 1980s, malaria and schistosomiasis are the focus of the first and second most numerous articles, respectively. It is likely that schistosomiasis will continue to be a barometer of progress in geospatial health sciences, when the attention of researchers is drawn to emerging issues, such as the efficiencies of integrated control of malaria, and the neglected tropical diseases (NTDs), which include schistosomiasis. The spatial, temporal, and spectral resolution of the satellite-based sensors, and the capabilities of computer-based models has led to an improved understanding of geographical areas, and how they can support the transmission of various infections. In addition, improved surveillance, risk-mapping, and access to large databases promise stronger possibilities for understanding the complex relationship between the environment and infection with regard to infections. For example, like so many other parasitic diseases, the interaction between the human definitive host and the intermediate snail host in schistosomiasis, depends strongly on ambient environmental variables, above all temperature. While the latter and accessibility to water, humidity, vegetation, and shade limit the snail distribution, the ambient temperature in the snail governs the speed of maturation of the infectious agent inside [21].

## 3. GEOHealth: Part of the Global Earth Observation System of Systems (GEOSS) 

Major scientific groups are interested in public health applications of the geospatial sciences, e.g., the Earth Science area of the National Aeronautics Space Administration (NASA) (http://www.nasa.gov/) has moved towards a strategic goal that includes the study of climate and environmental change and the potential impact on public health issues, such as infectious diseases, emergency preparedness and response (https://www.earthobservations.org/documents/cop/he_henv/2011032). NASA’s Public Health Program, chronicled by Luvall [22], is a growing part of the organization. In addition, the Group on Earth Observations (GEO) (https://www.earthobservations.org/geoss.php), an international agency with support from over 100 governmental departments, non-governmental organizations (NGOs), and scientific organizations has an interest in health, and so has the International Society for Photogrammetry and Remote Sensing (ISPRS) (http://www.isprs.org/). Yet another group, the International Medical Geology Association (MEDGEO) (http://rock.geosociety.org) has similar goals in its stated mission focused on the science dealing with the relationships between geological factors and health.

The growing availability of digital data for geospatial studies made possible by RS and resources from national space agencies, such as NASA in the USA, the French National Centre for space studies (Centre National d’Études Spatiales (CNES) [23], the European Space Agency (ESA) and the Japan Aerospace Exploration Agency (JAXA) [24] has led to the establishment of scientific teams that are interested in exploiting geospatial health applications for specific pursuits, e.g., public health research. Several dedicated journals have emerged, e.g., Geospatial Health (http://www.geospatialhealth.net) [25], the International Journal of Health Geographics (http://www.ij-healthgeographics.com) and Spatial and Spatio-Temporal Epidemiology (http://www.journals.elsevier.com/spatial-and-spatiotemporal-epidemiology/). The net result is that geospatial mapping and multidisciplinary modelling are becoming mainstream science in the health community at large. It is therefore of great potential value to cross-fertilize and reinforce linkages of diverse interest groups on health applications of the geospatial sciences. NASA programs promote linkages with the Group on Earth Observations (GEO) mission to build and utilize GEOSS (https://www.earthobservations.org/geoss.php) under the public health societal benefit area in which health scientists working on very different health issues can collaborate in the use of a standardized, interoperable, open-source global resource data portal. It would be expedient if the Earth observations health network (GEOHealth) within the GEOSS framework would gain stronger traction along the lines in the Box 1 below.

Box 1 **The GEOHealth Mandate.**

GEOHealth collaborates on activities relating to the GEO societal benefit area on Public Health and GEOSS, enabling the collaboration of governmental, inter-governmental, and non-governmental organizations to organize and improve mapping and predictive modelling of the distribution of infectious, vector-borne, and non-contagious diseases globally and make these data, information, and forecasts more accessible to policy and decision-makers, managers, experts, and other users. Such a network would progress from a Community of Practice to an Initiative and then a Flagship in the GEO work plan. This voluntary partnership would be guided by a steering committee comprising the key stakeholders, initially the ISPRS VIII/2 Working Group (http://www2.isprs.org/commissions/comm8/wg2.html) and the International Society of Geospatial Health (GnosisGIS) (www.gnosisgis.org) actively recruiting other organizations to join. GEOHealth draws on GEO’s data-sharing principles to promote full and open exchange of data, and on the GEOSS common infrastructure, to enable interoperability through the adoption of consistent standards. To assist both holders and users of health information to engage with GEOHealth, an active website would need to be established, containing links to information resources, activities, GEOHealth documents, meetings, and other resources that are relevant to the this mandate, including GnosisGIS, ISPRS VIII/2, the American Society of Tropical Medicine and Hygiene (ASTMH), and other groups interested in this endeavor to commit to the global vision of GEOHealth [26].

Is it possible to develop a dynamical 3-dimensional (3D) or even 4D (adding the temporal dimension) models of disease, such as bi-weekly global reports on the major endemic diseases? We are close to succeeding in this endeavor, facilitated by new satellite systems, big data, climatology advances, and novel sensors, such as the global precipitation model (GPM), the soil moisture active passive (SMAP), the Operational Land Imager (OLI), and the Thermal Infrared Sensor (TIRS), which replaces the Thematic Mapper Plus (ETM+) onboard the Landsat 8 satellite (summarized in Table 1). In addition, ESA’s Copernicus Sentinel mission includes a range of satellites carrying radar and multi-spectral imaging instruments for land, ocean, and atmospheric monitoring. Perhaps most importantly, the sub-meter resolution data now available from the image-focused Worldview 2, 3, and 4 satellites (https://www.digitalglobe.com/about/our-constellation) can provide community risk assessments. Adding to this, the elective value-added potential of the low-altitude sensors on drone airborne vehicles as a source of very high-resolution data collection within a user-set agenda [27,28].

Future NASA satellite missions, such as the Hyperspectral Infrared Imager or HyspIRI (http://hyspiri.jpl.nasa.gov/), will provide further enhanced capability to map vector-borne and other environmentally sensitive diseases, based on global hyperspectral visible and multispectral thermal data products (5-day, 60 m^2^ thermal and 19-day, 30 m^2^ hyperspectral repeat intervals) that will enable structural and functional classification of ecosystems, and the measurement of key environmental parameters, such as temperature and soil moisture. A new generation of sensors offer new capabilities, e.g., the ECOSTRESS instrument added to the International Space Station (ISS) on 29 June, 2018 (http://www.nasa.gov/jpl/nasas-ecostress) has started to monitor plant health using surface temperature measurements (and derived evapotranspiration values) with a 3-day to 5-day diurnal pair coverage, 38 × 57 m spatial resolution at varying times during the day due to the ISS orbit precession [22]. Timely adoption of these data resources in health surveillance and response systems will require close cooperation between NASA and public health scientists. In addition, very high-resolution satellite data collected by GeoEye-1, Worldview1-4, Quickbird-2 are available for both historical and current time periods from Digital Globe (https://www.digitalglobe.com/), a company recently acquired by Maxar Technologies (https://www.maxar.com/). These advances finally allow seamless mapping and modelling of diseases, not only at continental scales (1 km^2^) and local community-agricultural field scales (15–30 m^2^), but for the first time, also at the habitat-household scale (<1 m^2^) within individual communities.

A geospatial surveillance and response system resource for vector-borne disease in the Americas is currently being constructed using NASA satellite data, GIS, and ecological niche modelling to characterize the environmental and socioeconomic suitability, and the potential for the spread of selected endemic and epizootic vector-borne diseases in the Americas. The initial focus will be on developing prototype geospatial models on visceral leishmaniasis, an expanding endemic disease in Latin America, and models for dengue and other emerging *Aedes aegypti*-borne viruses (dengue, Zika, chikungunya) that have potential for epizootic spread from Latin America and the Caribbean to North America. We are planning to use the same resource data and modelling methods for surveillance and response systems for other vector-borne diseases, including schistosomiasis in the elimination phase. The GEOHealth concept would be a convenient way for incorporating the results into the interoperable, open-access standards of the GEOSS. Dissemination and training programs can then be implemented to promote geospatial mapping and modelling of vector-borne diseases, as envisioned in GEOSS. Implementation of GEOHealth requires, however, an initial effort to compile, design, and construct interoperable data structures that are anticipated to be useful for vector-borne disease surveillance and response systems based on the project investigators’ experience, literature reports and availability. In this way, all data will be resampled and projected in geographic formats compatible with other GEOHealth project data parameters, and available in ASCII form needed, e.g., for use in Maxent (https://www.gbif.org/tool/81279/maxent) [29] or Bayesian (OpenBUGS) mapping and modelling software. Data portal construction methods would be similar to that reported for a prior Pan American Health Organization (PAHO) project on mapping and modelling six neglected tropical diseases in Latin America and the Caribbean region [30]. To economize on the size of data storage requirements, data available for multiple years, e.g., the United States Geologic Survey (USGS) Landsat Legacy data would be acquired and archived in the data portal archive at 5-year intervals (2005, 2010, and 2015) with a step-by-step tutorial on how investigators can download additional data in the same format. Investigators would be able to examine data to evaluate usefulness using limited example data, with instructions on how to obtain similar additional complete data on specific time frames and scales, as needed from open-source archives linked to specified internet sites, e.g., the USGS Earth Resources Observation Systems (EROS) Data Center (https://eros.usgs.gov) which was established to study land change and to produce land change data products used by researchers, resource managers, and policy makers around the world. 

Data from the GEOHealth common resource data portal could then be used to demonstrate the feasibility of improved disease risk assessment models in prototype surveillance and response system models, as compared to previously reported models for vector-borne diseases that have a fundamentally different epidemiology, including schistosomiasis. We aim to develop geospatial development rate models that can simulate and display temporal progression (e.g., as 8-day snapshots) of vector–parasite life cycles and geospatial risk, based on comprehensive daily climate re-analysis data, night and day land surface temperatures (LST_night_, LST_day_), the normalized difference vegetation index (NDVI), the normalized difference moisture index (NDMI), and the normalized difference wetness index (NDWI) available from the Moderate Resolution Imaging Spectroradiometer (MODIS) on board the Terra and Aqua satellites The Visible-Infrared Imaging Radiometer Suite (VIIRS) will extend the MODIS program in the future. These data should used in the context of topography, land use, and population patterns. A major gap in the past has been environmental moisture data, which can now be addressed using newly available sensor systems data from SMAP, GPM, GOES-16, and ECOSTRESS (Table 1).

## 4. Mapping and Modelling NTDs in the Americas

**Figure 1:**
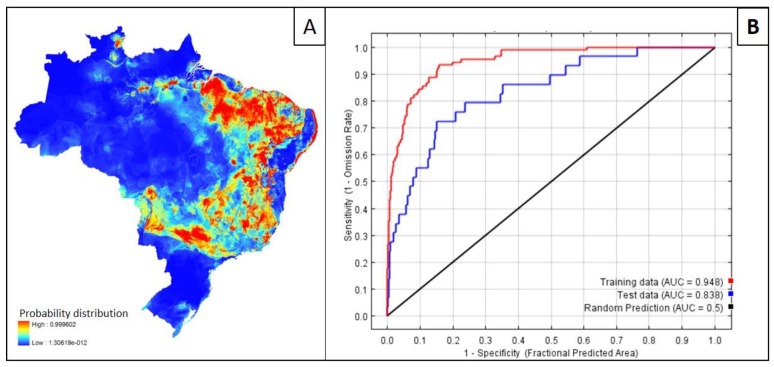
(**A**) Maxent-generated ecological niche model for predicting suitability for visceral leishmaniasis in Brazil based on the national surveillance program incidence data and Bioclim variables. (**B**) The accuracy of the model (0.838) was evaluated using Maxent by the area under the curve (AUC) of the receiver operating characteristic (ROC).

**Figure 2:**
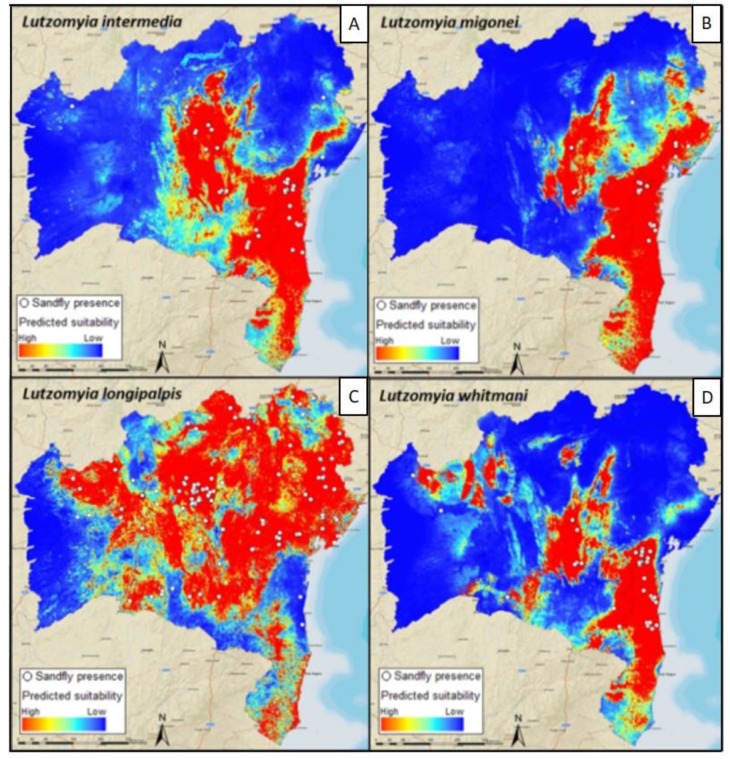
Maxent-predicted suitability for sand fly species of medical importance collected in Bahia state, Brazil. The output maps for the distribution of species incriminated as vectors of parasites that cause cutaneous leishmaniasis (**A**,**B**,**D**) and visceral leishmaniasis (**C**) were based on MODIS vegetation indices and Bioclim variables. Red areas indicate a higher suitability for vector occurrence.

**Figure 3:**
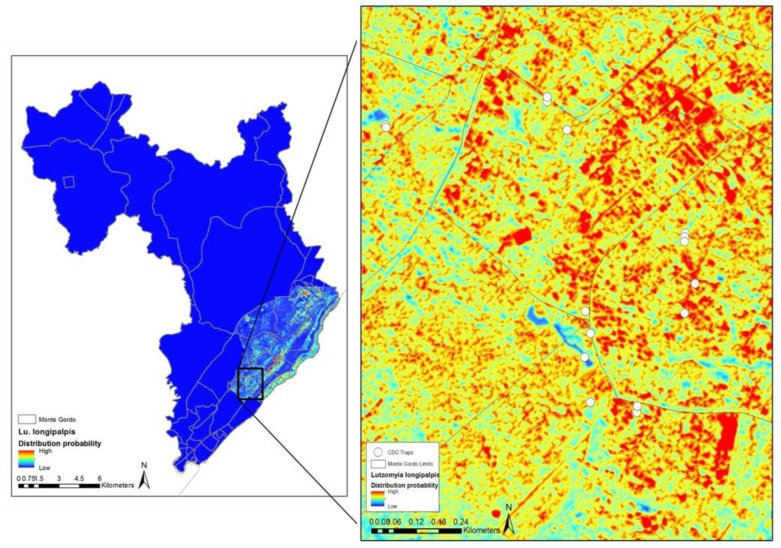
Maxent predicted the suitability for *Lutzomyia longipalpis* in Monte Gordo community-based on CDC trap data points and sub-meter spatial resolution WorldView2 imagery. The administrative boundaries of the municipality and districts (red lines) of Camacari, Bahia, Brazil are shown in the left panel. The predicted suitability of *Lutzomyia longipalpis* in Monte Gordo district is shown in the right panel based on CDC trap data and three vegetation indices derived from WorldView2 imagery, normalized difference vegetation index (NDVI), normalized difference soil index (NDSI), and normalized difference water index (NDWI). The inset box shows the model output and CDC trap locations. Highly suitable areas for the vector are shown in red.

Disease and vector occurrence data that are available at the national, state-wide and local community scale from earlier NTD studies in Brazil [30,31] funded by PAHO served as input for mapping disease and vector data using climate- and satellite-derived environmental data at the regional scale (1 km^2^ spatial resolution), the state-wide scale (15–30–60 m^2^ spatial resolution) and individual community scales (sub-meter spatial resolution). High-frequency climate and satellite sensor data can be made available in near real-time by access to Internet linkages to active program data. The selection of relevant environmental parameters to include in geospatial models, e.g., for visceral leishmaniasis, was based on results of regression analysis of disease and vector occurrence data, with variance inflation factor analysis to eliminate autocorrelation bias, according to the method of Mischler et al. [30]. Significantly associated Bioclim risk factors were included in Maxent as variables, and run with known vector and disease occurrence point data to develop probability risk surface maps that can be generated and incorporated as data layers in ArcGIS 10.6 mapping and modelling software. The relative contribution of each environmental variable to geospatial risk maps was evaluated by jackknife statistics, a part of the Maxent software package, to evaluate seasonality and relative risk as seen in Figure 1, Figure 2, and Figure 3 (from the doctoral thesis by Moara de Santana Martins [31]).

High resolution, biology-based geospatial mapping, and modelling methods can be developed and implemented by government agencies as the key to more rational, targeted control in surveillance and response systems for schistosomiasis that can interrupt and reverse the expansion to new endemic areas. Schistosomiasis in the elimination phase will require more sensitive case-finding diagnostic methods and satellite surveillance at the habitat–household resolution to pick up diminishing numbers of cases as control program success progresses. Sustained continuing surveillance programs are then required to prevent re-emergence. 

## 5. Schistosomiasis

Of all the vector-borne diseases, schistosomiasis was the topic of pioneer GIS studies done by Cross et al. [32], using the Landsat MSS (https://lta.cr.usgs.gov/MSS) satellite data and rainfall–temperature weather variables for geospatial risk assessment in the Philippines. Other early work was done as part of the Schistosomiasis Research Project (SRP) in Egypt, funded by the United States Agency for International Development (USAID), which showed that the Advanced very-high-resolution radiometer (AVHRR) temperature difference (dT) imagery could be used to map the risk of schistosomiasis in the Nile Delta [33], and that this was associated with the effect of the local hydrologic regime and shallow water table on the snail host–schistosome development cycle [34]. This work showed how well the GIS could address the classic concept of the ‘landscape epidemiology’, and Pavlovskii’s ‘essential nidality of disease’ concept [16] by virtue of its potential to match the relative suitability of various environments to the parasite life cycle and the transmission dynamics of host–parasite systems [35]. A more modern aspect is the attempt to predict the potential for future areas becoming endemic for schistosomiasis, due to the spread of the intermediate snail host, due to climate change [36].

### 5.1. Africa

With the African continent carrying the main burden off schistosomiasis by far, key countries in sub-Saharan Africa were selected for implementation of the ‘Schistosomiasis Control Initiative’ (SCI) (http://wwwsci-ntds.org), now the major control program in Africa. Basically a programme for the distribution of praziquantel, SCI, which applied GIS and RS to collect and record the cross-sectional national surveys on the distribution and intensity of schistosomiasis at the regional scale that were eventually used to guide optimal treatment strategies [37]. In this way, geospatial technology became linked to spatial information on climate, elevation, proximity to streams and water bodies activating innovative Bayesian geostatistical prediction models. Another activity was the Contrast project—a multi-disciplinary, 4-year alliance to optimize schistosomiasis control and transmission surveillance—that complemented the CSI by introducing an interactive agenda operating simultaneously at the molecular, biological, spatial, and social levels to identify risk factors governing the frequency and transmission dynamics of schistosomiasis [38]. The overall approach emphasized detailed knowledge of the distribution and abundance of snail hosts, bringing together existing information into a single database in an open-source Google Earth platform with Internet connection [39]. 

The accumulated experience on the transmission control of the Contrast program, facilitated by geospatial methods, contributed to the shift from an exclusive focus on morbidity control, to the adoption of the schistosomiasis elimination agenda in low-transmission countries [40]. In May 2012, the World Health Assembly passed a resolution calling upon member states to intensify schistosomiasis control and to initiate interventions towards local elimination [41]. This resulted in a focus on what was to be called the NTDs, and marked the start of a new era in the ambitious goal of elimination of schistosomiasis as a public health problem. The emergence of GIS, and access to Earth-observing satellite data as major tools in schistosomiasis research, and their integration into control strategies, has been excellently reviewed from the African scene by Mayangadze [42].

### 5.2. China and Southeast Asia

The International Symposium on Schistosomiasis, held in Shanghai, China [43] marked the beginning of geospatial tools for schistosomiasis control there. Using the NDVI, Land Surface Temperature (LST) and the Digital Elevation Model (DEM) extracted from MODIS and the Advanced Spaceborne Thermal Emission and Reflection Radiometer (ASTER) sensors onboard the Terra satellite, Zhu et al. [44] found that an ecological niche model integrated with NDVI, LST, elevation, slope, and distance from every village to its nearest stream could adequately predict snail habitats in the mountainous regions.

Even if snails can survive dry periods, water is the guarantee for their long-term survival and reproduction. The focus on snail habitats made GIS and RS necessary tools for the identification of land use, water bodies, vegetation, temperature, humidity LST, and vegetation and water indices [45]. These tools, including spatial statistics, are exceptionally useful for extracting and handling environmental data [46,47,48,49], and emphasized the importance of detailed updated information with wide geographical coverage. They further highlighted the advantages of RS technology over manual snail documentation, while Wang et al. [49] reported that a simple combination of the two indexes, normalized difference wetness index (NDWI) and normalized difference vegetation index (NDVI), made it possible to directly estimate the snail habitats quantitatively. This type of information should be useful for areas endemic for schistosomiasis japonica outside China, such as The Philippines and strongly limited endemic areas, such as those in the Sulawesi Island of Indonesia, where the exact borders of endemicity are difficult to settle. This could also be of value in the areas endemic for *S. mekongi* in Cambodia and Laos.

Schistosomiasis has a long history in The Philippines, with the disease ensconced in more than half of the country’s 78 provinces. Apart from the paper by Cross et al. [32], referred to above, relatively few papers on geospatial technology have appeared in the Philippines. Malone et al. [50], focusing on the implementation of a geospatial health infrastructure in Southeast Asia for the control of schistosomiasis, pointed out that health workers have not rapidly taken advantage of the widely available, low-cost spatial data resources for epidemiological modelling. Although the situation has since improved in China, the use of geospatial tools in The Philippines is still at the build-up stage [51,52]. 

### 5.3. Latin America

Adoption of geospatial approaches to schistosomiasis control in Latin America emerged in a similar timeframe as that in Africa and Asia. Analysis of the role of environmental factors for prevalence in representative Brazilian municipalities in a GIS shows that the population density and the duration of the annual dry period are the most significant determinants [53]. A follow-up study has given additional data on the temperature difference, and NDVI collected by the satellite-borne AVHRR sensor that was used for a GIS environmental risk assessment model for schistosomiasis in Brazil [54]. Joining the consensus in Brazil on the potential value of geospatial methods, Gazzinelli and Kloos [55] promoted use of spatial tools, while Guimarães et al. [56,57] reported the successful use of social, meteorological, and RS-derived digital elevation and NDVI data to delimit the risk for schistosomiasis at the municipality level in the state of Minas Gerais. 

A special issue of *Geospatial Health*, published in 2012, was devoted to geospatial applications for NTDs, including schistosomiasis, in South America and the Caribbean [58]. Of particular interest for the Brazilian distribution of schistosomiasis is the presence of two compatible snail host species: *Biomphalaria glabrata*, and *B. straminea* and that competitive selection makes *B. glabrata* dominate in irrigation systems, while *B. straminea* is more common in natural water sources [38,59]. Given the importance of socioeconomic and environmental risk factors in the persistence of transmission of NTDs, geospatial mapping and modelling was recognized early on, to be useful for the prediction of the distribution, and the prevalence of these diseases, and to identify areas where hotspots or disease overlap occurs. Significantly, the potential influence of climate change was often considered [16,35,60].

## 6. Healthy Futures

Concerns about the potential effects of impending climate change on vector-borne diseases was the focus of a major project funded in 2010–2014 by the European Commission’s 7th Framework (FP7)—Healthy Futures. The aim of his project was to contribute to reducing the future burden of three, water-related high-impact vector-borne diseases (VBD) in Africa—malaria, schistosomiasis, and rift valley fever (RVF). The project consortium comprised an inter-disciplinary group of climatologists, disease modellers, and experts in the environmental, health, and socio-economic sciences, together with staff in government health ministries in the East African Community (EAC).

A total of 15 institutions made up the consortium, located in 10 different countries, five African (Rwanda, Kenya, Uganda, Tanzania, South Africa) and five European (Ireland, Sweden, Austria, Italy, UK). VBD’s were expected to be sensitive to changes in environmental conditions, such as increased ambient temperature or changes in the timing and levels of rainfall associated with climate change. Dynamical simulation models were developed for each of the three targeted diseases based on data generated by the MODIS and the Tropical Rainfall Measuring Mission (TRMM) sensors. The climate surrogate data gathered covered the EAC at 1-km resolution at Earth surface. Model output risk maps were produced using ArcGIS software (http://www.esri.com/software/arcgis) based on current climate data and long-term climate change projections, as proposed by the Intergovernmental Panel on Climate Change (IPCC) in 2013. Predicted changes in the distribution and transmission patterns for malaria [61,62] schistosomiasis [63]), and RVF [64] were represented as maps covering the EAC, and decision support frameworks were developed for use by the scientific community and stakeholders in the EAC. 

Notably, an integrated, open-source Atlas based on the key results of the Healthy Futures project was produced [65]. This online resource provides information on past, present, and future conditions of the risk for malaria, schistosomiasis, and RVF and allows the exploration and visualization of results through web-based interactive tools. The Atlas embodies a guided access to information on climate change, the potentiality of disease occurrence, and population vulnerability, with respect to these three diseases in the EAC region through direct access to downloadable datasets and metadata integrated in the Healthy *Futures* Metadata Portal. Current available information can be directly accessed through the Healthy Futures website (http://www.healthyfutures.eu). 

Information can be queried based on three prime selection criteria: (i) the infectious diseases targeted; (ii) time, allowing for comparisons of current conditions with a range of future projections, while allowing access to information on historic outbreaks; and (iii) different components of risk. Future climate change projections based on two representative concentration pathways (RCPs) emission scenarios RCP4.5 (mid-level change) and RCP8.5 (high-level change) for each decade to 2100 throughout the EAC study area can thus be made available. Relative values of social vulnerability are mapped based on a range of indicators, such as susceptibility to disease (e.g., immunity, malnutrition, poverty, conflict, remoteness) and lack of resilience (e.g., education level, access to health facilities, number of dependents), while social and susceptibility indicators are weighted and combined in the form of a composite map indicator of geospatial risk [66]. The original Metadata Portal is hosted by the International Livestock Research Institute in Nairobi (ILRI). The metadata platform software used is freely available from ESRI (http://www.esri.com/software/arcgis/geoportal). The Portal uses the CSW (Catalogue Service for the Web) standard of the Open Geospatial Consortium (OGC) (http://www.opengeospatial.org), which makes it interoperable with other metadata portals and programs.

If successfully adopted and further developed, the Atlas will be among the first of its kind in geospatial health research to offer public health practitioners, scientists, and stakeholders a tool to enable the identification of likely VBD hotspots under different climate change scenarios at policy-relevant time-intervals over the coming century. Twelve articles emanating from the Healthy Futures ’Remote Sensing of Environment project’ were published in a special issue of Geospatial Health (http://www.geospatialhealth.net) in 2016. The emergence of GIS and Earth-observing satellite data as a major tool in schistosomiasis research, and their integration into real-world control strategies has been acknowledged by a large number of research teams. 

## 7. Future Potential

The NASA GEOSS program is currently divided geographically into an AfriGEOSS and AmeriGEOSS data resource effort, with the potential to add other defined regions, and it is these continental databases that will be used to develop GEOHealth applications, consistent with the NASA societal benefit area of public health. The members of the GEOHealth CoP (www.geohealthcop.org/) aim to both use and contribute GEOSS interoperable resource databases. 

The results of the Healthy Futures project provide an excellent candidate for developing public health applications within the AfriGEOSS program. The current NASA project ’GeoHealth: A Surveillance and Response System Resource for Vector Borne Disease in the Americas’ aims to contribute interoperable resource data and methods that are essential for vector-borne disease mapping and modelling in the Western Hemisphere, as part of the AmeriGEOSS program. Other data from Health and Air Quality Applications of the Applied Sciences program offer broader potential geospatial resources [66]. Global health databases, e.g., on schistosomiasis [38] and on sand flies [6] are emerging, that can be accessed for relevant health data to develop mapping and modelling applications, along with the addition of data from the existing literature, and results of new research projects in the future.

The virtual globe concept is not new, but the essential idea is now coming into its own. Many and various efforts in this direction have been made over the last 10–15 years. However, the field did not take off until user-friendly applications started to appear [67]. Intuitive technologies, such as Google Earth, enable scientists around the world to share data in a readily understandable fashion without the need for much technical assistance. In 2008, Elvidge and Tuttle felt that three-dimensional software modelling of the Earth leading to virtual globes would revolutionize Earth observation, data access, and integration [68]. Stensgaard et al. [38] and Yang et al. [18] used Google Earth for the management and control of vector-borne diseases, including schistosomiasis. The authors of this paper believe that the use of this approach can lead to a better understanding of the epidemiology and ecology of the neglected tropical diseases, including schistosomiasis, and other environmentally sensitive infectious diseases in the multidimensional environments in which they occur.

## 8. Conclusions

Currently available global geospatial data are underutilized by medical researchers. This may be due to the lack of the ability to bridge barriers to awareness, prioritization, or training deficits, which are needed for the interdisciplinary interaction of medical scientists with environmental scientists. The development of a GEOHealth platform would facilitate and encourage research to utilize and implement currently available geospatial analysis tools and new global data systems in surveillance and response systems for vector-borne diseases. 

Recently launched earth-observing satellite systems provide new opportunities to improve existing geospatial risk models that have already been effectively used to guide control programs for both filariasis [69] and soil transmitted helminths (STH) [70]. In particular, higher-resolution environmental analysis and the ability to evaluate life cycle drivers, as well as limiting moisture factors by new sensors such as SMAP and ECOSTRESS, are very promising tools for ecological niche modeling.

What is needed is an open-source, inter-operable platform that is freely accessible by the global health community to link public health workers with the most current potential earth observation resources from the geospatial sciences community. We propose that geospatial data resources from NASA and other national space agencies *can* be organized through a GEOSS virtual globe to make this possible. The vision, organization, and structure of the GEOHealth network is offered as a framework for initial effort as a vehicle for translational research, dissemination, and implementation in national public health systems in collaboration with GEO.

Given the strong progress on schistosomiasis elimination in several countries, China in particular, and the strong follow-up of the pioneer RS and GIS studies centered on this disease, it might well be used as model for the development and application of the new generation of space-based tools for NTD elimination.

## Figures and Tables

**Table 1:** Recently launched Earth-observing satellite resources for mapping and modelling GeoHealth applications.

Satellite Platform	Frequency	Swath	Sensor	Spatial Resolution	Applications/Comments
GPM ^a^ Launched Feb. 2014	Integrated multi-satellite retrievals (IMERGE) 0.5 hours	Dual-frequency Precipitation Radar (DPR) 125–245 km; Global Microwave Imager (GMI) 885 km	Core Observatory radar/radiometer system	1 km	Measures precipitation using a reference standard to unify measurements from a constellation of related research and operational satellites. Extends Tropical Rainfall Measuring Mission (TRMM) records
GOES ^b^ 16 Launched Nov. 2014	5–15 min	Full disk image of the Earth consisting of 22 swaths	Advanced Baseline Imager (ABI) with 16 bands	0.5–1 km–2 km	Meteorology; Geostationary orbit over the western hemisphere
Suomi-NPP ^c^ Launched Oct. 2011	Daily	3000 km	Visible-Infrared Imaging Radiometer Suite (VIIRS)	1 km	8-day Land Surface Temperature (LST) measurements for day and night. Extends MODIS ^d^, AVHRR ^e^
Soil Moisture Active Passive (SMAP)	3 h		L band Radar and Microwave Imager	3–10 km	Measures water content in the top 5 cm of the soil
Landsat 8 Launched Jan. 2013	16 days	185 km	Operational Land Imager (OLI), Thermal Infrared Sensor (TIRS)	OLI: Panchrom. = 15 m VIS-NIR-SWIR ^f^ = 30 m TIRS: thermal bands = 100 m	OLI and TIRS replace the Thematic Mapper (TM) and the enhanced Thematic Mapper Plus (ETM+) on previous Landsat satellites (Landsat legacy data has a continuous record since 1972
Sentinel 1 (A&B) A launched 2014 B launched 2015	12 days	250 km	C-band Synthetic Aperture Radar (C-SAR) Multi-spectral instrument	5 and 20 m	EU contribution to GEOSS with applications related to land, coastal water with respect to natural disasters, resources, environment, weather, seasonal forecasting and climate. Monitors plant growth and forests, changes in land cover marine and ecosystems through leaf chlorophyll and water content indexes
Sentinel 2 A launched 2015 B launched 2016	10 days	290 km	(MSI) with 13 channels in VIS-NIR-SWIR ^f^ Radar altimeter, micro wave	10, 20 and 60 m	
Sentinel 3 A launched 2015 B launched 2016	27 days	1270 km	radiometer, sea and land surface temperature radiometer	300 m	
Worldview 3 Aug. 2013 Worldview 4 Nov. 2016	<1 day	13.1 km	Pan, 8 Multi-spectral, 8 SWIR	Panchromatic = 31 cm Multispectral = 1.24 m	Optical data collection at the habitat-household level
International Space Station (ISS)	3 days	385–415 km	ECOSTRESS ^g^ Launched July 2018	38 × 57 m	Measures plant evapotranspiration (ET)

^a^ Global Precipitation Measurement (mission); ^b^ Geostationary Operational Environmental Satellites; ^c^ National Polar-Orbiting Partnership; ^d^ Moderate Resolution Imaging Spectroradiometer (MODIS); ^e^ Advanced Very High Resolution Radiometer; ^f^ Visual, Near Infrared and Short-Wave Infrared; ^g^ ECOsystem Spaceborne Thermal Radiometer Experiment on International Space Station. Table sources: https://earthdata.nasa.gov/user-resources/remote-sensors. Additional info pm Sentinel: https://directory.eoportal.org/web/eoportal/satellite-missions/c-missions/copernicus-sentinel-1.
